# Estimation of risk of neuropsychiatric adverse events from varenicline, bupropion and nicotine patch versus placebo: secondary analysis of results from the EAGLES trial using Bayes factors

**DOI:** 10.1111/add.15440

**Published:** 2021-04-22

**Authors:** Emma Beard, Sarah E. Jackson, Robert M. Anthenelli, Neal L. Benowitz, Lisa St. Aubin, Thomas McRae, David Lawrence, Cristina Russ, Alok Krishen, A. Eden Evins, Robert West

**Affiliations:** ^1^ Research Department of Behavioural Science and Health University College London London UK; ^2^ Department of Psychiatry University of California San Diego CA USA; ^3^ University of California San Francisco CA USA; ^4^ Pfizer Inc New York NY USA; ^5^ Formerly at GSK, Research Triangle Park NC USA; ^6^ Massachusetts General Hospital and Harvard Medical School Boston MA USA

**Keywords:** Bayes factor, bupropion, EAGLES, neuropsychiatric adverse event, nicotine patch, smoking cessation, varenicline

## Abstract

**Background and Aims:**

Analysed using classical frequentist hypothesis testing with alpha set to 0.05, the Evaluating Adverse Events in a Global Smoking Cessation Study (EAGLES) did not find enough evidence to reject the hypothesis of no difference in neuropsychiatric adverse events (NPSAEs) attributable to varenicline, bupropion, or nicotine patch compared with placebo. This might be because the null hypothesis was true or because the data were insensitive. The present study aimed to test the hypothesis more directly using Bayes factors**.**

**Design:**

EAGLES was a randomised, double‐blind, triple‐dummy, controlled trial.

**Setting:**

Global (16 countries across five continents), between November 2011 and January 2015.

**Participants:**

Participants were smokers with (*n* = 4116) and without (*n* = 4028) psychiatric disorders.

**Interventions:**

Varenicline (1 mg twice daily), bupropion (150 mg twice daily), nicotine patch (21 mg once daily with taper) and matched placebos.

**Measurements:**

The outcomes included: (i) a composite measure of moderate/severe NPSAEs; and (ii) a composite measure of severe NPSAEs. The relative evidence for there being no difference in NPSAEs versus data insensitivity for the medications was calculated in the full and sub‐samples using Bayes factors and corresponding robustness regions.

**Findings:**

For all but two comparisons, Bayes factors were <1/3, indicating moderate to strong evidence for no difference in risk of NPSAEs between active medications and placebo (Bayes factor = 0.02–0.23). In the psychiatric cohort versus placebo, the data were suggestive, but not conclusive of no increase in NPSAEs with varenicline (Bayes factor = 0.52) and bupropion (Bayes factor = 0.71). Here, the robustness regions ruled out a ≥7% and ≥8% risk increase with varenicline and bupropion, respectively.

**Conclusions:**

Secondary analysis of the Evaluating Adverse Events in a Global Smoking Cessation Study trial using Bayes factors provides moderate to strong evidence that use of varenicline, bupropion or nicotine patches for smoking cessation does not increase the risk of neuropsychiatric adverse events relative to use of placebo in smokers without a history of psychiatric disorder. For smokers with a history of psychiatric disorder the evidence also points to no increased risk but with less confidence.

## Introduction

The smoking cessation medications varenicline, bupropion and nicotine patch have been shown to improve smokers’ chances of stopping long term, but concerns have been raised about the safety of varenicline and bupropion with regard to neuropsychiatric adverse events (NPSAEs; e.g. suicidality and aggression) [1, 2]. Although meta‐analyses of clinical and observational studies have not substantiated these safety concerns [3, 4], the United States (US) Food and Drug Administration asked the manufacturers of these medications to conduct a large randomised trial to provide greater clarity on their potential safety risk [5] leading to the Evaluating Adverse Events in a Global Smoking Cessation Study (EAGLES; NCT01456936). This study compared the relative neuropsychiatric safety risk of varenicline, bupropion and the nicotine patch with placebo in smokers with and without psychiatric disorders. Analysed using a pre‐specified classical frequentist statistical approach, the study did not show a conventional statistically significant increase in NPSAEs attributable to varenicline, bupropion or the nicotine patch [5]. This paper calculates Bayes factors using the data from the EAGLES trial to assess whether the non‐significant findings using the frequentist approach for safety were the result of data insensitivity (i.e. there was a lack of power) or whether there was evidence for no effect; and, if the data were insensitive, whether the evidence tended to point in the direction of there being an effect or against it. This is important because when it comes to warnings about serious side effects of drugs, regulators, clinicians and patients need to have full information about the direction in which the available evidence points and not just the absence of clear evidence for an effect.

Traditionally, researchers have used the frequentist approach to assess the efficacy and safety of smoking cessation medications. This involves researchers formulating a hypothesis, collecting data to test this hypothesis and then calculating a test statistic (e.g. *t* test or χ^2^ test) and associated *P* value to identify whether there are differences among groups. The *P* value signals the extremeness of the data under the assumption that the null hypothesis is true and therefore generally, if it is <0.05 the researchers can conclude there is a significant difference among treatment groups. However, the *P* value only tells us the probability of a test statistic at least as extreme as the one observed occurring by chance. Therefore, a non‐significant effect (generally determined by a *P* value > 0.05) does not allow one to distinguish whether there is evidence of ‘no effect’ or the data are insensitive (i.e. there is a lack of power [6]). In other words, the *P* value does not tell us the probability of the null hypothesis being true. A more direct test of whether an effect is present involves the use of Bayes factors.

Conventional cut‐offs for the interpretation of Bayes factors are typically based on those set by Jeffreys [7] in the 1930s, with Bayes factors >3 representing moderate (substantial) to strong (extreme) evidence for the experimental hypothesis, values <1/3 representing moderate (substantial) to strong (extreme) evidence for the null hypothesis and values between 1/3 and 3 indicating that the data are insensitive [8, 9]. It has been estimated that only around 20% of non‐significant findings in randomised controlled trials in the field of addiction actually provide evidence of no effect, with the data generally being insensitive for the other 80% of trials [8].

However, there are also criticisms of the Bayes factor approach in that its calculation requires the specification of a predicted effect size and distribution, which this predicted effect size follows (known as a prior). Because of the subjective nature of this, it has been recommended that any conclusions are tested as robust to reasonable changes in the specification of the prior [10].

In summary, this paper reports a secondary Bayes factor analysis of the data from the EAGLES trial [5] to assess whether the null findings for differences in neuropsychiatric safety were indicative of there being no effect or data insensitivity. Two safety outcomes were assessed: (i) the primary composite neuropsychiatric endpoint, which covered moderate and severe NPSAEs; and (ii) the secondary composite neuropsychiatric endpoint, which covered severe NPSAEs only. Several sensitivity analyses were conducted to assess the robustness of prior assumptions used to calculate the Bayes factors.

## Methods

### Design

The EAGLES trial was a multinational (covering North America [USA, Canada], South and Central America [Argentina, Brazil, Chile and Mexico], Eastern Europe [Bulgaria, Russia and Slovakia], Western Europe [Denmark, Finland, Germany and Spain], Oceania [Australia, New Zealand] and South Africa), multicentre, randomised (1:1:1:1), double‐blind, triple‐dummy, placebo‐ and active‐ (nicotine patch, 21 mg/day with taper) controlled trial of varenicline (1 mg twice daily) and bupropion (150 mg twice daily). Treatment lasted 12 weeks with an additional 12‐week non‐treatment follow‐up. Full details of the trial design and randomisation can be found in the original publication of the trial results [5].

### Participants

Eligible participants were smokers, 18 to 75 years of age, with and without pre‐specified psychiatric diagnoses per the *Diagnostic and Statistical Manual of Mental Disorders*, Fourth Edition, Text Revision (DSM‐IV‐TR) [11], who smoked more than 10 cigarettes per day, had exhaled carbon monoxide concentration >10 p.p.m and were motivated to stop smoking. Participants were recruited from the investigators’ clinics, newspapers, radio and television advertising and fliers and posters. Those in the psychiatric cohort were diagnosed with either major depressive or bipolar disorders, anxiety disorders, psychotic disorders or borderline personality disorder. Participants had to be considered clinically stable for inclusion and considered by the investigator not to be at high risk of self‐injury or suicidal behaviour. Participants in the non‐psychiatric cohort had no confirmed history of DSM‐IV‐TR Axis I or II disorders.

Between November 2011 and January 2015, 8144 participants were recruited into the study. Data were analysed from 8058 smokers who took at least one dose of their study medication; 3984 in the non‐psychiatric cohort and 4074 in the psychiatric cohort. Table 1 shows the number of participants in each cohort who received the nicotine patch, bupropion, varenicline and placebo.

**Table 1:** Number of participants in each condition.

	*Non‐psychiatric cohort* *(n = 3984)*	*Psychiatric cohort* *(n = 4074)*
Varenicline	990	1026
Bupropion	989	1017
Nicotine patch	1006	1016
Placebo	999	1015

Overall, the study population included 3549 men (44%) and had an average age of 46.5 years. Smokers in the psychiatric cohort met DSM‐IV‐TR criteria for primary mood (*n* = 2882; 71%), anxiety (*n* = 782; 19%), psychotic (*n* = 386; 9%) and borderline personality disorders (*n* = 24; 1%).

### Outcomes

The primary endpoint was a composite measure based on post‐marketing reports of NPSAEs in smokers taking varenicline, bupropion and nicotine patch. It comprised 16 neuropsychiatric symptom categories (anxiety, depression, feeling abnormal, hostility, agitation, aggression, delusions, hallucinations, homicidal ideation, mania, panic, paranoia, psychosis, suicidal ideation, suicidal behaviour or completed suicide), which captured all volunteered, observed and solicited adverse events irrespective of whether they could be causally associated with the medication. To be included in the primary endpoint, adverse events had to be rated as moderate (some interference with daily functioning) or severe (substantial interference with daily functioning) for all adverse events except anxiety, depression, feeling abnormal and hostility, which were only included if rated as severe, given that these events are commonly associated with nicotine withdrawal. The secondary safety endpoint included the subset of all NPSAEs that were rated severe. Primary and secondary outcomes were coded as 1 if any symptoms were present and 0 if none were present.

### Randomisation

Eligible participants were stratified into a non‐psychiatric cohort and four sub‐cohorts in the psychiatric cohort based on their psychiatric primary diagnosis and geographical region. Participants were then randomised to receive varenicline (0.5 mg once daily for 3 days, 0.5 mg twice daily for 4 days, 1 mg twice daily for 11 weeks), bupropion (150 mg once daily for 3 days then 150 mg twice daily to end of week 12), nicotine patch (21 mg for 7 weeks starting at the beginning of the second week, 14 mg for 2 weeks, 7 mg for 2 weeks) or placebo in a triple‐dummy design for a 12 week treatment phase followed by 12 week non‐treatment phase. A randomisation administrator prepared the computer generated randomisation schedule used to assign participants to treatment using a block size of 8 (1:1:1:1) randomisation for each of the 20 diagnosis by region combinations.

### Procedure

Participants set a target quit date 1 week after randomisation to coincide with start of the full dose for varenicline and bupropion and the initiation of nicotine patch treatment. Smoking cessation counselling of at most 10 minutes was given at each face‐to‐face clinic visit (14, including the baseline visit). Telephone visits to determine smoking status (11 in total) were conducted during weeks when clinic visits were not scheduled. Emergence of adverse events was assessed at each face‐to‐face visit with open‐ended questions, direct observation and a semi‐structured Neuropsychiatric Adverse Events Interview (NAEI) conducted by trained interviewers to fully capture neuropsychiatric adverse events of interest [12]. General or psychiatric adverse events that met FDA requirements for serious adverse events (i.e. resulting in death, admission to hospital, substantial disability or life‐threatening event) were classified accordingly. Additionally, investigators assessed whether positive responses on the Columbia‐Suicide Severity Rating Scale (C‐SSRS) [13] and reports from participants’ family members or physicians were neuropsychiatric adverse events.

### Analysis

The analysis plan was preregistered on the Open Science Framework (https://osf.io/s7xda/). The primary analysis reported in this paper compared varenicline, bupropion, and the nicotine patch with placebo in the sample as a whole and subgroups with and without psychiatric disorders. This decision was based on the treatment by cohort interaction (*P* = 0·0652) reported previously [5].

Supporting information Table S1 gives Bayes factors for other comparisons of interest: varenicline versus the nicotine patch and bupropion versus the nicotine patch.

Bayes factors are the ratio of the likelihood of two hypotheses being correct given a set of data. When evaluating interventions, there are typically two competing hypotheses: the experimental hypothesis (H1; that the intervention had an effect) and the null hypothesis (H0; that it had no effect). Therefore, a Bayes factor is similar in form to a likelihood ratio [8, 14]:

(1) $$\text{Bayes factor}=\frac{\text{likelihood of data given}\;H_{1}}{\text{likelihood of data given}\;H_{o}}=\frac{P\left(\text{Data}|H_{1}\right)}{P\left(\text{Data}|H_{0}\right)},$$

which simply represents the probability of the data given the alternative hypothesis divided by the probability of the data given the null hypothesis.

For the present analysis, we extracted or derived from the original publication effect sizes (risk differences) and SEs for each of these 18 analyses; (i) the primary composite neuropsychiatric endpoint—varenicline versus placebo, bupropion versus placebo, and nicotine patch versus placebo in the sample as a whole and subgroups with and without psychiatric disorders; and (ii) the secondary composite neuropsychiatric endpoint: varenicline versus placebo, bupropion versus placebo, and nicotine patch versus placebo in the sample as a whole and subgroups with and without psychiatric disorders.

Risk differences and their standard errors were not calculated in the original publication for all comparisons. The following formulas were used in these instances:

$$\text{Risk difference}=p_{1}-p_{2}$$

(2) $$\mathrm{SE}=\sqrt{\frac{p_{1}\left(1-p_{1}\right)}{n_{1}}+\frac{p_{2}\left(1-p_{2}\right)}{n_{2}}},$$

where *p*_1_ and *p*_2_ are the probability of an adverse event in the active and control conditions and *n*_1_ and *n*_2_ are the sample sizes of the two conditions, respectively.

Bayes factors were calculated in R version 4.0.0 using code described by Dienes [15]. This approach requires the specification of an expected effect size (i.e. a plausible range of predicted values based on previous studies, judgement or clinical significance), the published effect size (e.g. risk difference) and the SE of this parameter. It assumes that the sampling distribution of the parameter estimate is Gaussian.

The predicted value for the effect size came from the sample size calculation included in the published EAGLES trial [5]. This specified an underlying event rate in the non‐psychiatric and psychiatric cohorts of 3.5% and 7.0%, respectively. It was determined that 2000 participants were needed in each treatment group to achieve sufficient power to detect an increase in the event rate of NPSAEs of 75% between treatment and placebo group within +1.59% or −1.59%. The calculated predicted effect sizes for the comparisons are given in Table 2.

**Table 2:** Predicted effect sizes from the sample size calculation.

	*Control*	*Treatment*	*Risk difference*
Non‐psychiatric group			
Total sample	1000	1000	–
Event	35	61.25	–
No event	965	938.75	–
Risk	0.035	0.061	0.026
Psychiatric group			
Total sample	1000	1000	–
Event	70	122.5	–
No event	930	877.5	–
Risk	0.070	0.123	0.053
Overall			
Total sample	2000	2000	–
Event	105	183.75	–
No event	1895	1816.25	–
Risk	0.053	0.092	0.039

We used a Gaussian (*μ*,*σ* ^2^) distribution where the population parameter values close to the mean are assumed to be more plausible than others. A default SD of mean/2 is often used [16]. Bayes factors were interpreted based on Jeffreys’ cut‐offs [7], which indicate the strength of evidence for or against the null hypothesis. More precisely, a Bayes factor <1/100 provides extreme evidence for the null hypothesis, 1/30 to 1/100 very strong evidence for the null hypothesis, 1/10 to 1/30 strong evidence for the null hypothesis, 1/3 to 1/10 moderate evidence for the null hypothesis and 1/3 to 1 anecdotal evidence for the null hypothesis. As a value between 1/3 and 1 indicates data insensitivity it can only provide suggestive evidence for the null hypothesis [9].

As a result of the subjective nature of the assumed prior distribution and predicted effect size, it has been recommended that any conclusions are tested as robust to reasonable changes in both the prior and assumed model [10]. Therefore, we also calculated a robustness region for each Bayes factor. The robustness region is a range of expected effect sizes that lead to the same qualitative conclusion as for the Bayes factor (i.e. good evidence for the alternative hypothesis if the Bayes factor is >3, good evidence for the null hypothesis if the Bayes factor is <1/3 and largely insensitive otherwise) [15].

We also assessed the robustness of the assumed model by conducting an additional sensitivity analysis using the Bayes Factor package in R [17] for the primary outcome. This package allows one to test the independence assumption for contingency tables with the prior based on the Dirichlet(α) distribution. It requires the specification of the sampling plan and ‘a’ that denotes the prior concentration [18]. The prior concentration indexes the expected deviation from the null hypothesis under the alternative and therefore was represented as a rate difference between the groups of interest based on the parameters specified in the sample size calculation. We selected an independent multinomial sampling plan where the columns reflecting group assignment were fixed.

### Role of the funding source

The funding sources for the original trial (EAGLES), Pfizer and GlaxoSmithKline, had no involvement in the study design of the present secondary analysis. The corresponding author (E.B.) and S.E.J. and R.W. conducted the analysis and prepared the initial draft of this manuscript. All authors had full access to all relevant EAGLES and secondary analysis data and critically revised the manuscript for intellectual content. The corresponding author had the final responsibility for the decision to submit for publication. Professional editorial assistance was provided by Engage Scientific Solutions and was funded by Pfizer. This involved formatting the paper for submission and collating responses and comments from all co‐authors.

## Results

As was reported in the original publication of the EAGLES trial results [5], no significant differences in the primary or secondary composite safety endpoints were found between varenicline, bupropion and the nicotine patch with the placebo condition, either overall or as a function of baseline psychiatric status. Tables 3 and 4 summarise the results of the Bayes factor analysis. See Supporting information Table S1 for full details of the values used in the Bayes factor calculations.

**Table 3:** Results of the Bayes factor analysis for the primary composite score (moderate to severe adverse neuropsychiatric treatment‐emergent events).

	*Risk difference from published study*	*SE of risk difference from published study*	*P value from published study*	*Bayes factor*	*Interpretation of Bayes factor*	*Risk difference robustness region* ^a^
Primary composite endpoint						
Overall						
Varenicline versus placebo	0.0029	0.0060	0.626	0.07	Strong evidence for the null	[0.015, ∞]
Bupropion versus placebo	0.0081	0.0062	0.193	0.23	Moderate evidence for the null	[0.032, ∞]
Nicotine patch versus placebo	0.0018	0.0060	0.760	0.06	Strong evidence for the null	[0.013, ∞]
Non‐psychiatric cohort						
Varenicline versus placebo	−0.0109	0.0060	0.072	0.08	Strong evidence for the null	[0.004, ∞]
Bupropion versus placebo	−0.0018	0.0067	0.792	0.08	Strong evidence for the null	[0.010, ∞]
Nicotine patch versus placebo	0.0008	0.0069	0.905	0.11	Strong evidence for the null	[0.013, ∞]
Psychiatric cohort						
Varenicline versus placebo	0.0160	0.0103	0.119	0.52	Insensitive/anecdotal evidence for the null	[0.00, 0.067]
Bupropion versus placebo	0.0176	0.0104	0.090	0.71	Insensitive/anecdotal evidence for the null	[0.00, 0.078]
Nicotine patch versus placebo	0.0029	0.0097	0.766	0.07	Strong evidence for the null	[0.021, ∞]

^a^ Indicates the range of expected effect sizes that lead to the same qualitative conclusion (i.e. evidence for the null [Bayes factor <1/3] or data insensitivity [Bayes factor between 1/3 and 3]).

**Table 4:** Results of the Bayes factor analysis for the secondary composite score (severe adverse neuropsychiatric treatment‐emergent events).

	*Risk difference from published study*	*SE of risk difference from published study*	*P value from published study*	*Bayes factor*	*Interpretation of Bayes factor*	*Risk difference robustness region* ^a^
Secondary composite endpoint						
Overall						
Varenicline versus placebo	−0.0015	0.0028	0.598	0.02	Very strong evidence for the null	[0.003, ∞]
Bupropion versus placebo	<0.0001	0.0030	0.990	0.02	Very strong evidence for the null	[0.005, ∞]
Nicotine patch versus placebo	−0.0005	0.0029	0.856	0.02	Very strong evidence for the null	[0.005, ∞]
Non‐psychiatric cohort						
Varenicline versus placebo	−0.0040	0.0025	0.103	0.05	Strong evidence for the null	[0.002, ∞]
Bupropion versus placebo	−0.0010	0.0030	0.750	0.03	Very strong evidence for the null	[0.004, ∞]
Nicotine patch versus placebo	−0.0020	0.0028	0.473	0.03	Very strong evidence for the null	[0.003, ∞]
Psychiatric cohort						
Varenicline versus placebo	0.0008	0.0051	0.869	0.03	Very strong evidence for the null	[0.010, ∞]
Bupropion versus placebo	0.0010	0.0051	0.850	0.03	Very strong evidence for the null	[0.010, ∞]
Nicotine patch versus placebo	0.0010	0.0051	0.848	0.03	Very strong evidence for the null	[0.010, ∞]

^a^ Indicates the range of expected effect sizes that lead to the same qualitative conclusion (i.e. evidence for the null [Bayes factor <1/3] or data insensitivity [Bayes factor between 1/3 and 3]).

### Primary outcome

For all but two comparisons, Bayes factors were <1/3, indicating that there was moderate to strong evidence for the null hypothesis of no difference in risk of NPSAEs. The robustness regions suggest that risk differences larger than 0.032 (3.2% difference in risk) for bupropion and 0.015 (1.5% difference in risk) for varenicline could be ruled out for all comparisons. The data were insensitive to determine whether a difference existed in the psychiatric cohort when comparing varenicline with placebo (Bayes factor 0.52) and bupropion with placebo (Bayes factor 0.71). However, these Bayes factors provided anecdotal evidence for the null hypothesis rather than the experimental hypothesis. The robustness regions suggest that we can rule out effects over 0.067 (7% difference in risk) and 0.078 (8% difference in risk) for varenicline and bupropion, respectively.

### Secondary outcome

For all comparisons, Bayes factors were <1/3, indicating that there was strong to very strong evidence for the null hypothesis of no difference in risk of severe NPSAEs. The robustness regions suggest that risk differences larger than 0.010 (1% difference in risk) for varenicline and 0.010 (1% difference in risk) for bupropion could be ruled out for all comparisons.

### Sensitivity analysis

Table 5 shows the result of the sensitivity analysis specifying a Dirichlet(α) rather than a Gaussian (*μ*,*σ* ^2^) distribution for the prior. Findings were similar to those of the primary pre‐planned analysis, with the data proving insensitive to determine whether a difference existed in the psychiatric cohort when comparing varenicline with placebo and bupropion with placebo. Evidence for the null hypothesis of no difference for all other comparisons was moderate to strong.

**Table 5:** Sensitivity analysis for the primary composite score (moderate to severe adverse neuropsychiatric treatment‐emergent events).

	*Number of events from published study*	*Number of non‐events from the published study*	*P value from published study*	*Bayes factor*	*Interpretation of Bayes factor*
Primary composite endpoint					
Overall					
Varenicline versus placebo	80/74	1936/1940	0.626	0.15	Moderate evidence for the null
Bupropion versus placebo	90/74	1916/1940	0.193	0.23	Moderate evidence for the null
Nicotine patch versus placebo	78/74	1944/1940	0.760	0.14	Moderate evidence for the null
Non‐psychiatric cohort					
Varenicline versus placebo	13/24	977/975	0.072	0.18	Moderate evidence for the null
Bupropion versus placebo	22/24	967/975	0.792	0.10	Strong evidence for the null
Nicotine patch versus placebo	25/24	981/975	0.905	0.10	Strong evidence for the null
Psychiatric cohort					
Varenicline versus placebo	67/50	959/969	0.119	0.36	Insensitive/anecdotal evidence for the null
Bupropion versus placebo	68/50	949/969	0.090	0.42	Insensitive/anecdotal evidence for the null
Nicotine patch versus placebo	53/50	963/965	0.766	0.18	Moderate evidence for the null

## Discussion

Secondary analysis of the EAGLES trial using Bayes factors indicated that the results largely provided evidence for the null hypothesis of no difference in risk of NPSAEs between those using varenicline, bupropion, or the nicotine patch for smoking cessation relative to those using placebo. Exceptions were comparisons of varenicline versus placebo and bupropion versus placebo for moderate to severe NPSAEs in a subgroup with diagnosed psychiatric disorders, where the inclusion of moderate events led to larger differences across treatment groups and, therefore, the data were insensitive to detect a difference in risk between conditions. However, even for these comparisons, large effects (greater than a risk difference of ~7–8%) could be ruled out and the Bayes factors analysis still favoured the null hypothesis. Additionally, Bayes factors analysis provided very strong evidence for the null hypothesis of no difference in risk of severe NPSAEs in the subgroup with psychiatric disorders, where the number of these composite endpoint events was virtually the same across all 4 treatment groups.

From a clinical perspective, these findings support the view that varenicline and bupropion do not meaningfully increase risk of moderate to severe NPSAEs [3, 4]. They provide additional information to enable policymakers, health professionals and smokers to make informed choices when deciding how best to address nicotine dependence.

From a methodological perspective, we believe that the use of Bayes factors in the presence of non‐significant findings is particularly important in safety studies to determine whether there is evidence for the null hypothesis of no effect or the data are insensitive. It should be noted that Bayes factors can also be used to quantify the evidence for an experimental hypothesis when a finding is significant (e.g. moderate, strong, very strong and extreme) and/or can be used as a stopping rule for data collection [8]. Calculation of a robustness region provides additional information on the size of effects that can be ruled out in the case of data insensitivity, allowing useful information to be extracted for results in situations where lack of statistical power is a concern.

The EAGLES trial had several limitations. First, the cohort of participants with diagnosed mood, anxiety, psychotic or borderline personality disorders included only those with conditions that were stable or had comorbid substance use disorders in remission. This might have led to selection effects and means that the findings might not translate to those who are untreated or have unstable symptoms. Second, as with any clinical trial, there are questions regarding ecological validity and whether the findings would translate to the real world where additional behavioural support is not always provided. Third, dropout occurred across treatment groups (see [5]), which could have affected outcomes, and the findings may not be applicable to lighter smokers because of the selection criteria. Fourth, this analysis did not consider non‐NPSAEs and therefore cannot rule out other adverse reactions. These were reported across treatments and cohorts and included insomnia (12% bupropion), nausea (25% varenicline), abnormal dreams (12% nicotine patch) and headache (10% placebo).

In conclusion, secondary analysis of the EAGLES trial results using Bayes factors provided moderate to very strong evidence that use of varenicline, bupropion or the nicotine patch for smoking cessation does not increase the risk of moderate to severe NPSAEs relative to use of placebo. The data were insensitive to confirm whether use of varenicline or bupropion increases the risk of moderate to severe NPSAEs relative to use of placebo among smokers with pre‐existing psychiatric conditions, but large differences could be ruled out, and there was very strong evidence of no increase in risk of NPSAEs that were rated severe.

## Clinical trial registration

Clinical trial registration #NCT01456936 (https://clinicaltrials.gov/ct2/show/NCT01456936).

## Declaration of interests

E.B. has received unrestricted funding from Pfizer. S.E.J. has no conflicts of interest to declare. N.L.B. has been a consultant to Pfizer and Achieve Life Sciences, companies that market or are developing smoking cessation medications. R.W. undertakes consultancy and research for and receives travel funds and hospitality from manufacturers of smoking cessation medications but does not, and will not, take funds from manufacturers of electronic cigarettes or the tobacco industry. He is also an advisor to the National Centre for Smoking Cessation and Training. T.M., D.L. and C.R. are employees of Pfizer. L.S.A. was an employee of Pfizer during the conduct and initial reporting of the EAGLES trial. A.K. was an employee of GlaxoSmithKline during the conduct and initial reporting of the EAGLES trial. A.E.E. reports advisory board service for Alkermes, Scientific Consultant service for Charles River Analytics (NIDA SBIR), Data Safety Board service for Karuna Pharmaceuticals (Data Safety Monitoring Board) and research grants to her institution from Brain Solutions, LLC (NIDA SBIR). R.M.A. provides consultancy to Pfizer and receives research support from Pfizer and Embera NeuroTherapeutics. EB and S.E.J. salaries are funded by Cancer Research UK.

## Author contributions

**Emma Beard**: Conceptualization; data curation; formal analysis; methodology; writing‐original draft; writing‐review & editing. **Sarah Jackson**: Conceptualization; methodology; writing‐review & editing. **Robert Anthenelli**: Writing‐review & editing. **Neal Benowitz**: Writing‐review & editing. **Lisa Aubin**: Writing‐review & editing. **Thomas McRae**: Writing‐review & editing. **David Lawrence**: Writing‐review & editing. **Cristina Russ**: Writing‐review & editing. **Alok Krishen**: Writing‐review & editing. **A.** **Eden Evins**: Writing‐review & editing. **Robert West**: Conceptualization; methodology; writing‐review & editing.

## Data‐sharing statement

On request, and subject to certain criteria, conditions and exceptions (see https :// www.pfizer.com/science/clinical‐trials/trial‐data‐and‐results for more information), Pfizer will provide access to individual de‐identified participant data from Pfizer‐sponsored global interventional clinical studies conducted for medicines, vaccines and medical devices; (i) for indications that have been approved in the USA and/or EU; or (ii) in programmes that have been terminated (i.e. development for all indications has been discontinued). Pfizer will also consider requests for the protocol, data dictionary and statistical analysis plan. Data may be requested from Pfizer trials 24 months after study completion. The de‐identified participant data will be made available to researchers whose proposals meet the research criteria and other conditions, and for which an exception does not apply, via a secure portal. To gain access, data requestors must enter into a data access agreement with Pfizer.

## Contributors

E.B., S.E.J. and R.W. had full access to all relevant data in EAGLES and take responsibility for the integrity of the data and the accuracy of the secondary data analysis presented. E.B., S.E.J. and R.W. conducted the analysis and prepared the initial manuscript draft. All authors critically revised the manuscript for intellectual content.

## Supporting information


**Table S1** Supporting Information.
